# The Impact of the Drying Process on the Antioxidant and Anti-Inflammatory Potential of Dried Ripe Coffee Cherry Pulp Soluble Powder

**DOI:** 10.3390/foods13071114

**Published:** 2024-04-05

**Authors:** Marta B. López-Parra, Irene Gómez-Domínguez, Maite Iriondo-DeHond, Esther Villamediana Merino, Vanesa Sánchez-Martín, Jose A. Mendiola, Amaia Iriondo-DeHond, Maria Dolores del Castillo

**Affiliations:** 1 Instituto de Investigación en Ciencias de la Alimentación (CIAL) (CSIC-UAM), C/Nicolás Cabrera, 9, Campus de la Universidad Autónoma de Madrid, 28049 Madrid, Spain; martab.lopez.parra@csic.es (M.B.L.-P.); ireneigd@hotmail.com (I.G.-D.); esthervillamedianamerino@hotmail.com (E.V.M.); vanesa.s@csic.es (V.S.-M.); j.mendiola@csic.es (J.A.M.); 2 Instituto Madrileño de Investigación y Desarrollo Rural, Agrario y Alimentario (IMIDRA), N-II km 38, 200, 28800 Alcalá de Henares, Spain; maite.iriondo@madrid.org; 3 Sección Departamental de Nutrición y Ciencia de los Alimentos, Facultad de Veterinaria, Universidad Complutense de Madrid, Av. Puerta de Hierro, s/n, 28040 Madrid, Spain; amaiairi@ucm.es

**Keywords:** antioxidant, coffee fruit pulp, digestion, inflammation, instant cascara, life cycle assessment, spray drying, water soluble powder

## Abstract

Coffee fruit cascara, which is the skin and pulp of the coffee cherry, has been authorized as a novel food for commercialization in the European Union. The present research assessed the feasibility of using spray drying to produce a soluble powder called instant cascara (IC), employing sun-dried ripe coffee cherry pulp as a raw material. Although there were no significant differences (*p* > 0.05) in the overall antioxidant capacity between the freeze-dried and spray-dried samples, after an in vitro simulation of the digestion process, the spray-dried sample was significantly (*p* < 0.05) more antioxidant. Both samples reduced physiological intracellular ROS and significantly decreased (*p* < 0.05) the secretion of the pro-inflammatory factor NO. Alkaloids and phenolic compounds were detected in intestinal digests. In conclusion, spray drying is a good technique for producing IC as its use does not affect its properties and causes less environmental impact than freeze drying, as calculated by life cycle assessment. Sensory analysis did not show significant differences between the commercial beverage and the IC beverage in the adult population. IC at 10 mg/mL was significantly less accepted in adolescents than the commercial beverage. Future work will include the reformulation of the IC beverage at 10 mg/mL, which has antioxidant and anti-inflammatory potential, to increase its hedonic acceptance in all consumer segments.

## 1. Introduction

In April 2021, the dried coffee cascara, the outer skin and pulp obtained from the wet processing of coffee berries, from *Coffea arabica* L. and *Coffea canephora* Pierre ex A. Froehner was designated a safe traditional food by the European Food Safety Authority (EFSA) [1]. EFSA approved dried coffee cascara as a novel food in 2022 and concluded that beverages produced by infusing it in water will be available for consumption [2]. In a world facing population growth, climate change, resource depletion, and global crises, we must reduce waste to protect the environment and improve living conditions. Upcycling food waste as novel ingredients is one mechanism to achieve the more sustainable and healthy diets needed to guarantee food and nutrition security for all. This approach is in line with the 17th Sustainable Development Goal of the United Nations, which aims to end hunger, achieve food security, improve nutrition, and promote sustainable agriculture, as well as ensure sustainable consumption and production patterns.

Products based on coffee cascara are already available on the European market. Most of the beverages on the market are liquid drinks made from coffee cascara and come in both sweetened and unsweetened variants, some of which are carbonated and mixed with different flavours [3,4,5]. Another commercially available cascara-based product is kombucha [6,7]. Tabifruit is a Spanish coffee cascara product commercialized by Supracafé [8]. Methods have been patented for extracting nutrients from the entire coffee cherry or a portion thereof (US 8,603,563 B2). These include the preparation of powders from whole quick-dried sub-ripe coffee cherries. Coffeeberry^®^ cascara, a beverage-ready powder rich in antioxidant polyphenols derived from upcycled coffee fruit, is commercialized in the USA [9]. However, to the best of our knowledge, there are no commercially available soluble powders from dried ripe coffee cherry pulp, obtained after a careful selection of ripe Arabica cherries, pulping, sun drying, ionization, and drying after water extraction by either spray drying or freeze drying.

Previous studies carried out by our research group have proposed the use of the coffee fruit cascara to produce instant cascara (IC) by freeze drying (FD) [10]. Drying is an essential process for the long-term preservation of foods and food ingredients and allows them to be distributed and stored more easily and economically [11]. In this context, there are two basic methods to convert a liquid cascara extract into dry powder, which are the most popular and practiced techniques for drying in the food industry: freeze drying (FD) and spray drying (SD) [12].

FD requires freezing the liquid first and then exposing it to a vacuum, in such a way that water changed into ice crystals is removed as vapours by sublimation, yielding the dried product [12,13]. SD is one of the most widely used technologies in the food industry due to its low cost and flexibility and is established as the most economical process for commercial production. In SD, the liquid flows by hot air into a preheated drying chamber (dried in situ), dried particles and exhaust stream are separated by passing through a cyclone separator, and the dried microparticles are collected in the collector. FD is considered a gentle method used for drying products with heat-sensitive compounds since it keeps the initial functional properties of these components almost intact, providing a product with a high aroma quality. The main limitation of SD is the high temperatures used, with the possibility of losing certain low-boiling-point aromatic compounds. On the other hand, FD requires a longer drying time due to the lower heat transfer rate to the core of the frozen extract, and it is also energy-intensive and costly due to the low temperature and pressure employed. Furthermore, an FD product is not as portable as an SD powder due to its higher water activity and lower bulk density, suggesting freeze-dried powders have a more open and porous structure [12,13,14,15,16].

Inflammation and oxidative stress are processes linked to different chronic pathologies, such as cancer, diabetes, neurodegeneration or arthritis, and aging. It has been described that oxidative damage by Reactive Oxygen Species (ROS) contributes to carcinogenesis, and elevated ROS levels have been found in colorectal cancer, among others [17]. In addition, increased pro-inflammatory factors contribute to DNA damage, which is associated with tumour development [18]. In Alzheimer’s disease, the increase in ROS induces the expression of pro-inflammatory genes, and oxidative stress and pro-inflammatory factors decrease insulin secretion and disrupt insulin signal transduction, respectively [18]. The impact of the drying process on the antioxidant and anti-inflammatory potential of dried ripe coffee cherry pulp soluble powders has not been investigated and it is of great interest. These powders may act as carriers of antioxidant and anti-inflammatory polyphenols during digestion. To achieve this goal, the beverage should be prepared from the soluble powder at a specific dose in order to be accepted by consumers. This information is very relevant for demonstrating the potential marketability of the product and the assignment of health claims to soluble powders.

The present study aimed to compare the bioactive profile, antioxidant and anti-inflammatory properties, and environmental impact of dried ripe coffee cherry pulp soluble powders produced by either spray drying (SD-IC) or freeze drying (FD-IC). A sensory analysis, business model, and operations and investment plan for the commercialization of the soluble powders were also developed. The final goal of the investigation was to contribute to the sustainability of the coffee industry and to better understand the health-promoting potential of newly authorized novel foods, such as dried ripe coffee cherry pulp, which can potentially reduce the risk of global chronic diseases.

## 2. Materials and Methods

### 2.1. Chemicals and Reagents

The chemicals used were of analytical grade. Folin–Ciocalteu reagent, 2,2′-azino-bis(3-ethylbenzothiazoline-6-sulfonic acid) diammonium salt (ABTS), 2,2-diphenyl-1-picrylhydrazyl (DPPH), HCl, NaOH, potassium persulfate (K_2_S_2_O_8_), potassium phosphate dibasic (K_2_HPO_4_), sodium phosphate monobasic (NaH_2_PO_4_), chlorogenic acid, sodium carbonate, sulphanilamide, n-1-(naphthyl)ethylenediamine-dihydrochloride, α-amylase from human saliva (A0521), porcine gastric pepsin (P6887), porcine pancreatin (P1625), porcine bile extract (B8631), cholestyramine, and methanol were purchased from Sigma-Aldrich (St. Louis, MO, USA).

For cellular experiments, Dulbecco’s modified Eagle medium (DMEM), L-glutamine, antibiotics (penicillin and streptomycin), and trypsin were purchased from Gibco Laboratory (Invitrogen Co., Grand Island, NY, USA). Foetal bovine serum (FBS) was purchased from Hyclone (GE Healthcare, Chicago, IL, USA). 3-(4,5-dimethylthiazol-2-yl)-2,5-diphenyltetrazolium bromine (MTT), 2,7-dichlorofluorescein diacetate (DCFH-DA), lipopolysaccharide (LPS) from *Escherichia coli* O55:B5, tert-butyl hydroperoxide (tBOOH), vitamin C, and nitric oxide (NO) were purchased from Sigma-Aldrich (St. Louis, MO, USA).

The water used was purified by a Milli-Q system (Merck Millipore, Burlington, MA, USA).

### 2.2. Raw Materials

#### 2.2.1. Raw Cascara

The coffee fruit cascara, the skin and pulp of the coffee cherry, used in the present study was of the Arabica species, Tabi variety, obtained from Colombia and kindly provided by Supracafé S.A. (Móstoles, Madrid, Spain). Cherries were selected by Multiscan CoffeeXcan, a specially designed system for sorting and inspecting coffee cherries by combining advanced vision and X-ray technology, which allows very efficient sorting by degree of maturity and internal quality. Coffee fruit cascara was obtained from wet coffee cherry processing, dried for 21 days in the sun, and subjected to a sanitation process by ionization (beta beams) (IONISOS IBÉRICA, Tarancón, Cuenca, Spain).

Analysis of the microbiological quality and presence of mycotoxins in the raw material was carried out according to Commission Implementing Regulation (EU) 2022/47 [2] to guarantee the safety of the product.

#### 2.2.2. Commercial Product (Tabifruit)

Cascara infusion 100% Arabica was provided by Supracafé S.A. (Madrid, Spain).

#### 2.2.3. Preparation of Dried Ripe Coffee Cherry Pulp Soluble Powders

Aqueous extracts from dried ripe coffee cherry pulp were obtained by the aqueous extraction of 75 g of raw material in 1.5 L of water at 100 °C for 10 min, and then filtering (250 μm). FD-IC was obtained in a Delta 2-24 LSCplus freeze dryer (CHRIST, Ottobeuren, Germany) at 0.019 mBar and 21 °C. SD-IC samples were either prepared directly from the filtered extract or concentrated by ultrafiltration, before the spray-drying process, to increase the yield of production of the powder. After the extraction, the resulting infusion was divided into two fractions, and the fractions were concentrated by ultrafiltration to obtain two individual solutions with 9.5 and 15 °Brix, respectively. The concentration procedure was carried out in dynamic mode, employing a 2 kDa cut-off membrane, constant transmembrane pressure of 2.5 bar, and room temperature. Subsequently, the unconcentrated sample and two concentrated solutions underwent spray drying to produce the corresponding soluble powders. SD was performed in a MiniSprayDryer S-300 apparatus (Büchi, Barcelona, Spain) at an air inlet temperature of 170 °C, and the outlet air temperatures varied from 90 to 110 °C. The microbiological quality of the products was assessed to ensure their safety before further investigation following the same methods used for the raw material (Section 2.2.1).

##### Product Yield Calculation

A higher yield means a higher profit; therefore, it is an important indicator for the industry [19]. Two yield values are reported: (1) the yield of the whole soluble powder process using the amounts (g) of initial raw material (dried ripe coffee cherry pulp) used for extraction and final soluble powder (Equation (1)); (2) the yield of the drying process based on the experimental and theoretical values of the obtained soluble powder, where the theoretical or expected amount of final product is estimated using the concentration (°Brix) of the infusion to dry and the weight (g) of the whole volume of infusion to dry (Equation (2) [20]):

(1) $$Yield\;of\;the\;whole\;soluble\;powder\;process\;\left(\%\right)=\frac{g\;of\;soluble\;powder\times 100}{g\;of\;raw\;material}$$

$$theoretical\;value\;(g\;of\;expected\;soluble\;powder)=\frac{Concentration\;of\;infusion\;\left({}^{\circ}Brix\right)\times weight\;of\;infusion\;to\;dry\;(g)}{100}$$

(2) $$Yield\;of\;the\;drying\;process\;\left(\%\right)=\frac{experimental\;value\;(g\;of\;soluble\;powder)\times 100}{\;g\;of\;expected\;soluble\;powder}$$

### 2.3. Sensory Analyses

Sensory analyses of instant cascara, at 10 and 4 mg/mL, were carried out and compared with a commercial product made from coffee fruit cascara (Tabifruit) following the instructions of the manufacturer.

Consumer acceptance of the beverages was evaluated in two consumer segments: 74 adults (23 men and 51 women, age range between 22 and 53 years old) and 63 adolescents (27 men and 36 women, age range between 15 and 18 years old) (total sample n = 137). Participants were recruited at Instituto de Investigación en Ciencias de la Alimentación (CIAL, CSIC-UAM) as part of a science activity within the “Science Week” program for schools. The assays were carried out following the indications established in the ISO 8589:2007 standard. The participation of the consumers was voluntary, and no monetary compensation was given. Beverages were coded with a three-digit number and presented in random order to prevent first-order and flavour carryover effects. Consumer acceptance of the beverages was assessed using a nine-point hedonic scale that ranged from “1—dislike extremely” to “9—like extremely”. The attributes evaluated included “overall liking”, “visual appearance”, “smell”, and “taste”. In addition, a five-point just-about-right (JAR) scale was used to measure the appropriateness of the level of the following attributes: “colour intensity”, “sweetness”, “coffee flavour”, “bitterness”, and “fruity flavour”. The JAR scale ranged from 1 (“not enough at all”) to 5 (“far too much”). Liking and JAR data were used for a penalty analysis to study the relation between the rankings on the JAR scale and the results of the liking scores for the different attributes. Hedonic analyses of the beverages were performed using a one-way ANOVA with Tukey’s test for assessing differences between samples. Calculations were conducted in the SPSS 25.0 statistical package (SPSS Inc., Chicago, IL, USA) and XLStat-Sensory version 2018.6. Differences were considered significant at *p* ≤ 0.05. The results from the hedonic test are presented as the mean rating for each beverage attribute ± standard deviation.

### 2.4. Physicochemical Parameters

#### 2.4.1. Moisture Content

The moisture content of the powders was determined using an MA 35 moisture analyser (Sartorius, Göttingen, Germany). Analysis was carried out in duplicate for two samples of each powder. The results are expressed as a moisture percentage (%). The results were normalized by taking into account the moisture content of the samples.

#### 2.4.2. pH

The pH of IC beverages (10 mg/mL) was measured using an MP 230 pH meter (Mettler Toledo, Barcelona, Spain). The beverages were analysed in triplicate.

#### 2.4.3. Soluble Solids Analysis

The soluble solids content (°Brix) of the IC beverages (10 mg/mL) was determined using a model PR-32α digital refractometer (ATAGO, Tokyo, Japan).

#### 2.4.4. UV-Vis

The UV–visible spectra of the IC beverages were obtained using a microplate reader (BioTek Epoch 2 Microplate Spectrophotometer, Winooski, VT, USA). The absorption spectra of the samples were recorded in the UV-Vis range from 200 to 700 nm [21]. UV-Vis spectra provide fast information regarding the modification of food composition due to food processing. The structure of bioactive compounds such as caffeine, chlorogenic acid, and melanoidins may be affected during the drying process, changing the typical UV-Vis spectrum of the sample.

#### 2.4.5. Colour

Colour measurement was carried out with a SPECORD 210 Plus Reflectance Integrating Sphere (Analytik Jena, Jena, Germany) with illuminant D65 and an observer placed at 10°. The colour of the powders and the IC beverages (10 mg/mL) was analysed using the CIE (Commission International de l’Éclairage) colour space L*a*b* as numerical values, representing luminosity and colour parameters. The colour coordinates L*a*b* indicate brightness, green-red colouring, and yellow-blue colouring, respectively [22].

#### 2.4.6. Total Phenolic Content (TPC)

For TPC analysis in the IC beverages (10 mg/mL), the Folin–Ciocalteu method was carried out in a micro-method format [23]. The reaction was initiated by the addition of 10 μL of sample or standard and 150 μL of the Folin solution to a 96-well plate. After 3 min of incubation at 37 °C, 50 μL of sodium carbonate (283 mM) was added. The samples were incubated for 2 h and absorbance was measured at 735 nm using a UV–visible spectrophotometer (BioTek Instruments, Winooski, VT, USA). Chlorogenic acid (CGA, 0.00–0.7 mg/mL) was used as standard. The results are expressed as mg of CGA eq. per gram of dried sample (mg of CGA/g of dried sample). A duplicate sample was made, and measurements were performed in triplicate.

#### 2.4.7. Analysis of Phenolic Compounds by HPLC-QTOF

SD-IC and FD-IC phenolic compound identification was carried out using HPLC equipment (Agilent 1200, Santa Clara, CA, USA) equipped with a coupled degasser (G1322A), quaternary pump (G1311A), thermostated column module (G1316A), thermostated automatic injector (G1367B), and diode array detector (G1315B). It was coupled to a mass spectrometer (Agilent G6530A Accurate Mass QTOF LC/MS) with an electrospray atmospheric pressure ionization (ESI) source with JetStream technology. The control software was MassHunter Data Acquisition (B.05.00) and MassHunter Qualitative Analysis (B.07.00). Samples and standard solution were injected (20 µL for extracts and 2 µL for digested) in a ZORBAX Eclipse XDB-C18 column (150 mm × 4.6 mm × 5 µm) at 40 °C. The digests required a 1:10 dilution. The solvent systems were 0.1% formic acid (solvent A) and 0.1% formic acid diluted in acetonitrile (solvent B). The elution gradient was as follows (time, % of solvent A): 0 min, 95%; 20 min, 85%; 30 min, 70%; 35 min, 50%; 37 min, 95%; 45 min, 95%. Analysis was carried out in duplicate for each sample.

#### 2.4.8. Analysis of Methylxanthines by HPLC-QTOF

The identification of the SD-IC and FD-IC methylxanthines by HPLC-QTOF was performed with the HPLC equipment (Agilent 1200) described above. The samples and standard solution were injected (20 µL for theobromine and theophylline, 1 µL for caffeine) in a ZORBAX Eclipse XDB-C18 column at 40 °C. The extracts and their digests were diluted 1:100 and 1:10, respectively, for injection. The elution gradient was as follows (time, % of solvent A): 0 min, 95%; 20 min, 85%; 30 min, 70%; 35 min, 50%; 37 min, 95%; 45 min, 95%. Analysis was carried out in duplicate for each powder.

### 2.5. The Bioactivity of Compounds Present in the Digests

#### 2.5.1. In Vitro Oral–Gastrointestinal Digestion

Powders can be used as ingredients (food and beverage) and dietary supplements. Therefore, the digestion of powder provides useful information regarding the dose that ought to be used in different formulations to obtain the expected biological effect. This information is relevant for the association of health claims with the powders and derived products. The conditions of digestion reproduce human physiological conditions. Powders were digested in duplicate mimicking human digestion conditions, as described by Hollebeeck et al. [24] and modified by Martinez-Saez et al. [25], in three phases (oral, gastric, and duodenal). Briefly, 1.2 g of sample (SD and FD) was weighed and 7 mL of MiliQ distilled water was added, adjusting the pH to 6.9. The enzyme α-amylase prepared in PBS was added (0.433 mL per sample), and samples were placed at 37 °C for 5 min and 350 revolutions per minute (rpm). After 5 min, the reaction was stopped by a pH change with the addition of 1 M HCl. For the gastric phase, the samples were adjusted to pH 2; the enzyme pepsin, previously prepared in HCl, was added; and the samples were incubated at 37 °C for 90 min and 350 rpm. After this time, the reaction was stopped by a pH change through adding 1M NaHCO_3_. Finally, in the duodenal phase, the samples were adjusted to pH 7, and 1 mL of pancreatin enzyme and 883.2 mg of bile per sample were added. The samples were incubated at 37 °C for 150 min and at 350 rpm. At the end of the incubation time, the reaction was stopped by an abrupt change in temperature. The soluble fraction of the digest was treated with 10% activated resin (cholestyramine) for one hour at room temperature under agitation to remove residual bile salts from the samples. After the removal of cholestyramine by centrifugation, the samples were aliquoted for further analysis.

#### 2.5.2. Antioxidant Capacity

##### ABTS^•+^ Scavenging Assay

The trapping capacity of cationic free radicals was evaluated using the method of radical ABTS^•+^ bleaching described by Re et al. [26] following the procedure described by Martinez-Saez et al. [25] for its use in a microplate. ABTS^•+^ stock solution was prepared by mixing the ABTS^•+^ radical and potassium persulfate and incubated for 16 h at room temperature. Subsequently, the ABTS^−+^ working solution was prepared by diluting in a stock solution of 5 mM sodium phosphate buffer at pH 7.4 to 1:75 (*v*/*v*) and adjusted to an absorbance of 0.7 ± 0.02 at 734 nm. Aqueous solutions of CGA (0.0–200 µM) were used for calibration. Absorbance was measured in a microplate using a UV–visible spectrophotometer (BioTek Instruments, Winooski, VT, USA). Analyses were performed in triplicate. The results are expressed as mg of CGA eq. per gram of dried sample (FD-IC or SD-IC) (mg of CGA/g of dried sample).

##### Determination of Radical Scavenging Capacity against DPPH

DPPH free radical scavenging capacities were determined by the DPPH^•^ assay. In the presence of an antioxidant, the DPPH^•^ radical can accept an electron or hydrogen radical to become a stable molecule without dimerization [27]. When this occurs, the violet colour of DPPH^•^ decays, and the absorbance change can be followed spectrophotometrically at 517 nm [28].

In short, a stock solution of DPPH (0.125 g/L) in methanol was initially prepared. Subsequently, from this stock solution, the daily DPPH solution was made at a final concentration of 0.05 g/L, with adjustments made to attain an absorbance reading of 0.7 ± 0.02 at 517 nm. Each well of a 96-well microplate contained 273 µL of DPPH and 7 µL of sample (final volume: 280 µL). CGA was used as a reference standard (0.00–1.4 mmol/L). After incubation for 60 min in the dark, the absorbance of the reaction mixture was measured at 517 nm using a UV-Vis spectrophotometer (BioTek Instruments, Winooski, VT, USA). A duplicate IC beverage (10 mg/mL) was made, and measurements were performed in triplicate. The results are expressed as mg of CGA eq. per gram of dried sample (mg of CGA/g of dried sample).

##### Intracellular Reactive Oxygen Species (ROS) Formation

The human colon cancer cell line (Caco-2) was from the Bioanalytical Techniques Unit (BAT) of the Instituto de Investigación en Ciencias de la Alimentación (CIAL) (Madrid, Spain). CCD-18 cells, from human normal colon tissue, were purchased from the Centro de Instrumentación Científica (CIC) of the Universidad de Granada, Spain. The cells were cultivated as a monolayer in DMEM, supplemented with 10% (*v*/*v*) heat-inactivated FBS, 50 U/mL of penicillin, 50 µg/mL of streptomycin, and 1% (*v*/*v*) L-glutamine, under standard conditions (37 °C and 5% CO_2_) in a humidified incubator (BINDER CB series 2010, Tuttlingen, Germany). 

Prior to the determination of ROS, the effect of different concentrations of SD-IC and FD-IC on cell viability was measured by the MTT assay. This colorimetric method is based on the reduction of the tetrazolium ring of MTT by the mitochondrial enzyme succinate dehydrogenase, yielding a blue formazan product that can be measured spectrophotometrically (570 nm), allowing the determination of the mitochondrial functionality of the treated cells [29,30]. In each assay, untreated cells were considered as a negative control and DMSO (50%) was used as a positive death control.

The assessment of basal intracellular ROS levels was conducted using the procedure outlined by Iriondo-DeHond et al. [31] by measuring the fluorescence intensity of the DCFH-DA probe, which was proportional to the amount of ROS formed. Tert-butyl hydroperoxide (tBOOH) 1 mM was used as a positive oxidation control and vitamin C (100 µg/mL) was used as an antioxidant control. Subsequently, an MTT assay was conducted to standardize the data based on the cell count per well. The experiments were carried out in duplicates and two different cell passages. Intracellular ROS formation was calculated as follows:

% ROS formation=FluorescenceSampleMTT AbsorbanceSample FluorescenceControlMTT AbsorbanceControl ×100

#### 2.5.3. Anti-Inflammatory Properties

The anti-inflammatory potential of SD-IC and FD-IC was assessed by measuring the nitric oxide (NO) production in murine macrophages (RAW 264.7) as described by Benayad et al. [32]. RAW 264.7 cells were seeded on a 96-well plate (8 × 10^4^ cell/well) and cultured in DMEM with 4.5 g/L of glucose, 10% *v*/*v* of FBS, 1% *v*/*v* of L-glutamine, and 1% *v*/*v* of antibiotics for 24 h (37 °C, 5% CO_2_). Then, cells were pretreated with samples (0.1 mg/mL, 150 µL/well) diluted in medium without FBS for 24 h (37 °C, 5% CO_2_), and then were treated with LPS (1 µg/mL) + sample (0.1 mg/mL) (150 µL/well) and incubated for another 24 h. After incubation period, 100 µL of cell supernatants and standard curve (NO from 0 to 10 µg/mL) were transferred to another 96-well plate and mixed with 100 µL of Griess reagent (1% (*w*/*v*) sulphanilamide and 0.1% *w*/*v* n-1-(naphthyl)ethylenediamine-dihydrochloride in 2.5% *v*/*v* H_3_PO_4_). The mixtures were allowed to incubate at room temperature in the absence of light for 15 min, after which the absorbance was recorded at 550 nm in a BioTek Epoch 2 Microplate Spectrophotometer (Winooski, VT, USA). Control cells were used in each assay. Analyses were performed in duplicate and two different cell passages.

### 2.6. Environmental Impact Quantification

**Figure 1:**
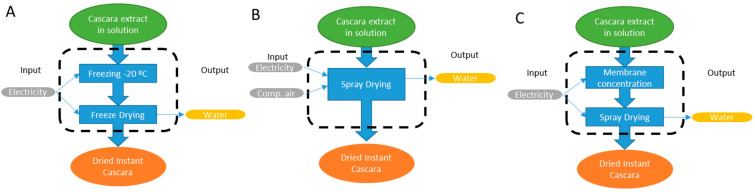
System boundaries (limited by dashed line) considered for the LCA calculation to obtain 1 kg of instant cascara (functional unit) in the three considered processes. (**A**) Freeze drying, (**B**) only spray drying, (**C**) spray drying after membrane concentration.

The environmental impact of the two drying processes was analysed by LCA (life cycle assessment) procedure using the software SimaPro 8 (Pré Consultants B.V., Amersfoort, The Netherlands). ILCD (International reference Life Cycle Data system) was chosen as the calculation method [33], using the SimaPro option “ILCD 2011 Midpoint + V1.08/EU27 2010, equal weighting”. The two drying methods were compared using the final yield obtained. In this sense, 1 kg of dried instant cascara was chosen as the functional unit (FU) to which the environmental impact categories were normalized. The system boundaries and flowchart considering the three drying processes to perform LCA analysis can be seen in Figure 1. A gate-to-gate approach was considered, where the upstream and downstream steps linked to the drying processes were not included.

### 2.7. Business Model and Operations and Investment Plan

In order to determine the commercial viability of the product mentioned in this work, different tools were used. A literature review was carried out to propose technological solutions to sustainable problems in the coffee industry. Finally, a business model and an operations and investment plan, which lists all investments and their corresponding costs associated with the enterprise to achieve the financial objectives of the business, were proposed to launch the idea to the market.

Consistent with Nielsen and Lund [34], “The business model is […] the platform which connects resources, processes and the supply of a service which results in the fact that the company is profitable in the long term. This definition emphasizes the need to focus on understanding the connections and the interrelations of the business and its operations so that the core of a business model description is the connections that create value”. A business model is a management strategy by which companies could have a “structured way for sustainable business thinking by mapping the purpose, opportunities for value creation across the network, and value capture (how to generate revenue) in companies” [35].

According to the business model developed in this work, two customer segments could be differentiated. The buyers, who will place the product on the market to be bought by the second segment, and the users, who will be every consumer, regardless of gender and who are concerned about their health. The value proposition is to offer a product with a sustainable competitive advantage that is differentiated from the rest of the products on the market, representing a healthy drink due to the high fibre content of cascara, its low caffeine content, and its potassium, magnesium, and vitamin C levels [36].

### 2.8. Ethics Statement

The sensory acceptance study of IC was granted a favourable ethical opinion from the Spanish National Council Ethics Committee on the 30th of July 2021 (internal reference: 114/2021). The study was conducted following the ethical standards that were laid down by the 1964 Declaration of Helsinki. All the participants gave written informed consent to participate in the study and they were aware of the possibility of withdrawing from the study at any time they desired. This activity is part of the project entitled “New beverages from by-products of coffee for optimal health of the brain-intestinal axis (COFFEE4BGA)” (ref. no.: PID2019-111510RB-I00), whose main researchers are Dr. del Castillo and Dr. Abalo.

### 2.9. Statical Data Analysis

All data are reported as the mean ± standard deviation. A t-Student test or one-way analysis of variance (ANOVA), applying the Tukey test, was used to determine significant differences between samples (*α* ≤ 0.05). Analyses were performed using the Statgraphics Centurion 18 program (Statgraphics Technologies, Inc., The Plains, VA, USA).

## 3. Results and Discussion

The dried ripe coffee pulp used as a raw material for the preparation of soluble powders was microbiologically safe. The levels of mycotoxins (ochratoxin A and aflatoxins B1, B2, G1, and G2) and microorganisms (aerobic plate count, total yeasts and moulds, *Enterobacteriaceae* and *Salmonella*) were within the limits described in the Commission Implementing Regulation (EU) 2022/47 [2].

The yield of the water-soluble product obtained by FD (24.3%) was similar to that previously reported by Iriondo-DeHond et al. [10] (20%). The drying process did not significantly affect (*p* > 0.05) the yield of production of the powders. Data support the feasibility of SD (yield = 21.1%), avoiding the addition of carriers to produce IC.

As expected, the concentration of the samples before drying increased the yield of production, these being 11.2% and 7.8% for those solutions of 9.5 and 15 °Brix, respectively. Concentration reduces moisture content and consequently lowers the energy required for water evaporation. However, concentration increases the viscosity of the product to be dehydrated, affecting the function of the machine and the drying yield [37]. The frequency of the cleaning of the machine should be increased to avoid its damage.

Yields of drying calculated using data of total soluble solids content (°Brix), as reported in [20], were 62.4% for FD and 54.1% for SD, respectively. Another study reported a different value for spray-dried juçara fruit (a black-violet berry) pulp (66%) without the application of a carrier [38]. This value was calculated as the relationship between the total solids obtained after drying and the total solids in the initial solution.

Commonly, the spray drying of fruit juices or infusions requires the use of a carrier, product concentration, or modification of specific process parameters, including low air humidity and outlet air temperature, to achieve an adequate yield [39]. The use of spray drying without the addition of carriers, as is proposed in the present paper, offers the advantage of preserving the concentration of bioactive compounds while avoiding alterations in the natural colour and flavour of the food product. In addition, it can lead to cost savings by eliminating the need for carriers. Therefore, the SD-IC was made in the same way as spray drying instant coffee, without a carrier [40].

### 3.1. Sensory Analysis

The results of the sensory analysis of IC beverages, at 4 mg/mL and 10 mg/mL, and Tabifuit are shown in Table 1.

**Figure 2:**
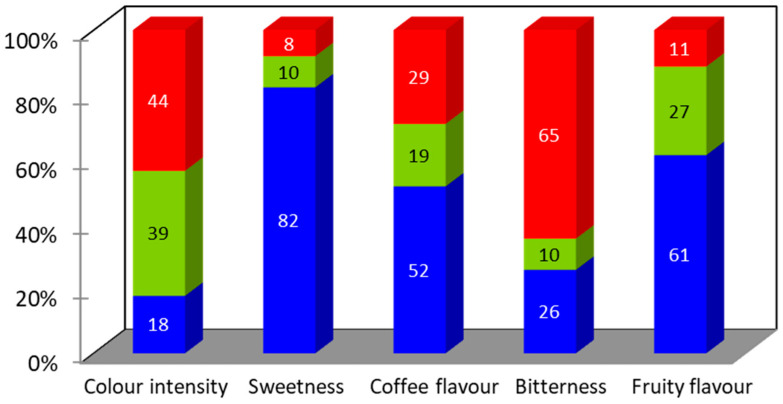
Just-about-right (JAR) scale percentages of responses regarding attributes of IC 10 mg/mL in adolescents. Blue: too low, green: just about right, red: too high.

For the adult population, no significant differences (*p* > 0.05) were observed among the three samples studied, for any of the attributes considered in the analysis. In the case of adolescents, IC 4 mg/mL and Tabifruit beverages were significantly better accepted than IC 10 mg/mL (Table 1). The JAR scale results for the adolescent population and the IC 10 mg/mL beverage are shown in Figure 2. More than 80% of the adolescents reported that IC 10 mg/mL was too low in “sweetness”. Several studies confirm that sweet taste is innate and evident among children around the world. This preference for sweetness remains high during childhood and early adolescence, beginning to decline progressively in mid-adolescence until reaching adult stages [41]. The penalty analysis showed the mean drop in liking scores for the attributes that had a significant negative effect (*p* < 0.05) and an occurrence higher than 20% of cases. These parameters identified that too little “fruity flavour” produced a significant (*p* < 0.01) mean drop in the overall liking of 2.09. Too little “sweetness” and too much “bitterness” resulted in a mean drop in the overall liking of 0.79 and 1.89, respectively, although it did not produce a significant negative effect (*p* > 0.05). In order to increase the acceptance of this beverage in this population, a reformulation of the product is contemplated. The addition of hypocaloric sweeteners or the combination with other fruit extracts will be considered.

### 3.2. Physicochemical Parameters

The physical parameters of IC obtained by freeze drying and spray drying are shown in Table 2.

The moisture content of FD-IC (5.32%) was significantly higher (*p* < 0.05) than that found for the powder obtained by spray drying, also called SD-IC (3.71%), which confirmed that this technique was less effective at removing water [42]. Moisture content impacts on the quality and the shelf life of foodstuffs, being one of the environmental variables that can speed up quality decay [43,44]. The moisture content of the powders was higher than that determined for other similar powder products, which showed moisture contents lower than 3% [45,46,47]. However, the values are within the accepted range for atomized powders [48]. Quek et al. [45] reported that in the SD process, at a higher inlet temperature, the heat transfer rate to the particle is greater, which provides a greater driving force for moisture evaporation. Consequently, powders with a reduced moisture content are formed; nevertheless, increasing the inlet temperature could influence the composition or properties of the resulting powder.

The SD-IC beverage was significantly (*p* < 0.05) less acidic than that obtained by freeze drying, suggesting the loss of acid compounds during its processing. The pH value of FD-IC was in line with that previously reported by Iriondo-DeHond et al. [10]. Acidity can be attributed to phenolic compounds and organic acids contained in coffee fruit cascara, such as chlorogenic, caffeic, ferulic, gallic, syringic, vanic, coumaric, and hydroxybenzoic acids [49]. Generally, phenolic compounds are more stable under acidic conditions compared to alkaline conditions, and this prevents their oxidation and degradation [50,51,52]. The difference in pH in the products obtained by the different drying techniques could be explained by the possible volatilization of some acid compounds due to the high temperatures used in spray drying [53]. Pua et al. [54] identified a total of 151 volatile compounds in extracts of five coffee cascara samples, of which phenolic and their derivatives represented approximately 1 and 7%. In addition, the optimum pH for accentuating flavour notes and preventing bacterial growth is between 3 and 4 [22]. Low moisture content and acid pH may contribute to extending the shelf-life of the products [43,44,55].

The total solid content of beverages prepared with the same concentration (10 mg/mL) of both powders was of the same order of magnitude (*p* > 0.05). Murlida et al. [56] reported 0.4 °Brix for Arabica coffee cascara tea prepared using five times less cascara to make the infusion than the one prepared in the present investigation, suggesting a lower concentration of dissolved solid components, which may consist of total sugar, amino acids, vitamins, pigments, protein, and organic acids [56,57].

On the scale of lightness (L), when values are closer to 100, the product is lighter, and values closer to 0 represent a darker product. Colour is a quality indicator and consumer preference criterion. The spray-dried powder showed a higher L* value and lower moisture content than the freeze-dried powder. This finding agreed with data previously reported by other authors for soluble coffee powders produced using the same technologies described here [15]. Moisture content has been linked to changes in colour parameters. Nara Tobaldini et al. [58] concluded that colour parameters can be applied for a preliminary assessment of moisture content in yerba mate leaves.

As shown in Table 2, the liquid sample beverages (10 mg/mL) showed a different behaviour than the powders. The freeze-dried beverage showed a higher L value than that prepared by dissolving spray-drying powder. These results suggest a higher effective concentration of coloured compounds in the spray-dried beverage associated with its lower moisture content in comparison with the one from freeze-dried soluble powder. As reported by Iriondo-DeHond et al. [10], melanoidins generated during the drying process of ripe coffee cherry pulp appear to be responsible for the colour of IC. The two beverages showed a similar UV–visible absorption spectrum (Table 2). The maximum absorption in the range of 273 and 326 suggests the presence of caffeine and phenolic compounds [10]. The results are in line with those shown in Table 3.

### 3.3. Bioactivity of Compounds Present in Digests

#### 3.3.1. Analysis of Methylxanthines and Phenolic Compounds by HPLC-QTOF

The composition of methylxanthines and phenolic compounds (Table 3) in FD and SD coffee cascara extract was studied. The total content of bioactive compounds of both samples was 17.23 and 41.47 mg/g of dried sample for FD-IC and SD-IC, respectively. Significant differences in the total content of bioactive compounds between the two samples were found (*p* < 0.05). The total value of methylxanthines was lower in FD-IC than in SD-IC. The total values of isoflavones and hydroxycinnamic acids were higher in SD-IC than in FD-IC, while anthocyanins were not found in the former. The samples were prepared from the same coffee cascara.

Alkaloids are secondary metabolites mostly present in plants, to which different biological properties have been attributed [59]. Within this group, caffeine, theobromine, and theophylline are alkaloids derived from purine, known as methylxanthines. They are produced in various vegetal species, such as coffee, tea, and cocoa [60]. Methylxanthines are noted for their antioxidant potential, reducing oxidative DNA damage [61]. Caffeine was found at a significantly higher concentration (*p* < 0.05) in SD-IC than FD-IC; however, theobromine and theophylline were detected at lower concentrations in SD-IC. This could be due to the higher temperature sensitivity of the two compounds. Ref. [62] found that the rate of dehydration of theophylline changed in an air temperature-dependent manner. Another research work demonstrated that theobromine concentration increased with temperature up to 170 °C and then decreased, probably due to its degradation to other compounds [63].

The total content of phenolics in food and their antioxidant potential vary and are influenced by numerous intrinsic and extrinsic parameters, such as the temperature used, light exposure, sample pretreatment, enzymes, proteins, storage, and oxygen [64]. Phenolic compounds are the most prevalent secondary metabolites, including flavonoids, phenolic acids, and coloured anthocyanins, for which numerous biological activities have been described (antioxidant, anti-aging, anti-inflammatory, and antiproliferative) [65]. Isoflavones and hydroxycinnamic acids were not significantly affected by the higher temperatures of the spray-drying process. Mangiferin was the most abundant isoflavone and 3-O-caffeoylquinic acid was the major hydroxycinnamic acid.

The values of mangiferin and epicatechin were significantly higher in SD-IC. Mangiferin possesses antioxidant capacity and the ability to regulate several important inflammatory pathways, thus protecting a wide range of physiological disorders [66]. Mangiferin has been detected in the leaves of the coffee plant in similar quantities to those found in our study for SD-IC (662.84 µg/g). Differences in its concentration in the powders may be associated with differences in temperatures used in both processes [66,67].

Focusing on hydroxycinnamic acids, p-coumaroylquinic acid was unique in these compounds detected at a significantly lower concentration (*p* < 0.05) in the sample SD-IC compared to FD-IC, which could be due to the increase in temperature. Michalska et al. [68] described that the 3-p-coumaroylquinic acid value decreased when comparing FD to other drying methods at higher temperatures. 5-O-Feruloylquinic acid and 4,5-di-O-caffeoylquinic acid were detected in higher amounts in the SD-IC, with the difference being statistically significant (*p* < 0.05).

A different behaviour was observed in the anthocyanin family. Cyanidin-3-O-glucoside and cyanidin-3-O-rutinoside were identified in FD-IC, while they were not detected in SD-IC. These compounds were previously detected in fresh coffee cascara, with a higher content of cyanidin-3-O-ruthinoside than cyanidin-3-O-glucoside [69]. Their absence in SD-IC may be a result of the high spray-drying inlet temperatures [70].

The profile of bioactive compounds of the digested fraction is shown in Table 4.

Concentrations of 867.55 and 663.27 µg of caffeine/mL of digest were found in the samples corresponding to FD-IC and SD-IC, respectively. Soares et al. [71] described that in espresso coffee capsule beverages, the values of caffeine were 1600 and 825 µg/mL after in vitro digestion (intestinal phase). The concentration of caffeine in FD-IC was similar to the latter value in espresso coffee capsule beverages; however, in SD-IC, the caffeine value was lower. Caffeine represents the major alkaloid recovered from coffee fruit cascara samples and it is considered one of the most active molecules in coffee. It is absorbed in the stomach and the small intestine; its pharmacology is complex, depending on the doses used and the different studies; and it can cross the blood–brain barrier [72,73]. This bioactive compound has a positive effect on the central nervous system, helping to improve physical and cognitive performance, with neuroprotective, anti-inflammatory, and immunomodulatory properties [72,73]. Normal plasma caffeine concentrations are 10–50 µM [74], and physiological concentrations of caffeine are normally less than 70 µM [75]; thus, the supraphysiological caffeine concentration of the samples would be active. Based on the EFSA information, “the maximum concentration of caffeine in infusions produced using the novel food could be up to 600 mg/L of drink, a concentration comparable to those in coffee beverages” [76]. Therefore, the caffeine concentrations of the samples would be within the safe range.

In parallel, after digestion, no significant differences (*p* > 0.05) were found in the concentration of bioaccessible theobromine and theophylline for both digested fractions. Catechin hexoside was detected in amounts that are in line with compounds identified in coffee by-products by other authors [77].

3-O-Caffeoylquinic acid resulted in the most abundant phenolic compound identified in the digested fraction, with the value obtained for SD-IC digest being significantly higher (*p* < 0.05). Other authors already reported the stability of this compound after simulated digestion [78]. Various effects have been attributed to chlorogenic acids: neuroprotective, anti-inflammatory, microbiota modulatory, antidiabetic, antihypertensive, or hypocholesterolaemic [73]. The physiological concentrations of chlorogenic acid are in the range from 2 nM to 6 µM [79]. The values of chlorogenic acid in the digests were 11- and 13-fold higher than 6 µM in the FD-IC and SD-IC, respectively; thus, chlorogenic acid concentrations in the samples would be active. The content of chlorogenic acid in FD-IC and SD-IC were lower than the values of espresso coffee from single-dose capsules after in vitro intestinal digestion (420–720 µg/mL) [80].

No significant differences (*p* > 0.05) in the quantification of total phenolic compounds in the digests were found.

The presence of anthocyanins was not detected in the digest of FD-IC and SD-IC. Temperature and pH are factors that may induce changes or a degradation of their structures [81]. Liang et al. [82] reported that the intestinal environment (pH = 7.4) causes a change in the molecular structure, thus generating phenolic acid compounds. Moreover, in other research work, anthocyanins were not detected after in vitro gastrointestinal digestion in coffee pulp aqueous extract [83].

#### 3.3.2. Overall Antioxidant Capacity

The antioxidant capacity analysed as the total phenolic content (TPC) and radical scavenging capacity against DPPH and ABTS is shown in Table 5. The scavenging of ABTS^•+^ by the studied instant cascara was higher than that of DPPH· radicals. This may be because ABTS^•+^ cation radicals are more sensitive to high-molecular-weight phenolics and more reactive than DPPH· radicals, wherein the reaction of ABTS^•+^ radicals with antioxidants is completed within 1 min [84].

The beverages produced by both dehydration processes and prepared at the same concentration (10 mg/mL) showed no significant differences (*p* > 0.05) in antioxidant capacity (Table 5). The high temperatures used in spray drying do not seem to affect the antioxidant properties of the coffee fruit cascara, which is considered a potential source of phenolic compounds with antioxidant properties and the potential to improve human health [22,85].

The antioxidant capacity of the digested extracts is shown in Table 6.

The FD-IC and SD-IC digests had a quantifiable antioxidant response. The digest obtained from the simulated human digestion of SD-IC showed a significantly higher (*p* < 0.05) antioxidant capacity than that corresponding to FD-IC. This could indicate that during the digestion of the SD-IC, specific compounds that significantly increase (*p* < 0.05) its antioxidant capacity were released compared to the FD-IC, as is the case of chlorogenic acid [73]. The data showed a statistically significant difference (*p* < 0.05) in the values of this acid, with them being higher in the SD-IC. To the author’s knowledge, this is the first time that the antioxidant capacity and phenolic compound profile of a digested coffee cascara fraction has been evaluated.

##### Intracellular Reactive Oxygen Species (ROS) Formation

**Figure 3:**
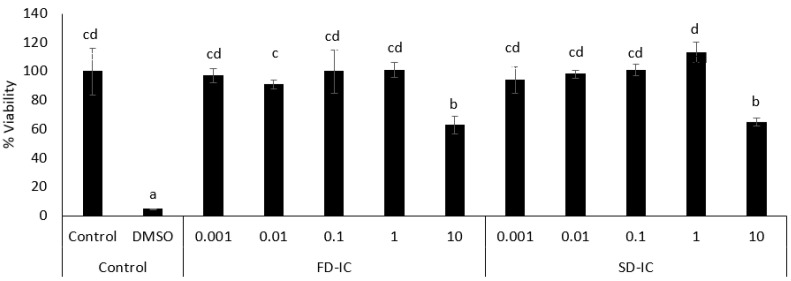
Effect of FD-IC and SD-IC on Caco-2 cell viability. Control refers to untreated cells and DMSO (50%) was used as a death control. Different letters indicate significant differences between samples (Tukey’s test; *p* < 0.05).

Figure 3 shows the viability of Caco-2 cells after 24 h of incubation with FD-IC and SD-IC at different concentrations (0.001 to 10 mg/mL). The viability was not affected by treating the cells with the two studied samples at concentrations between 0.001 and 1 mg/mL. Based on these results, a dose of 0.1 mg/mL was selected to perform an intracellular ROS formation assay.

**Figure 4:**
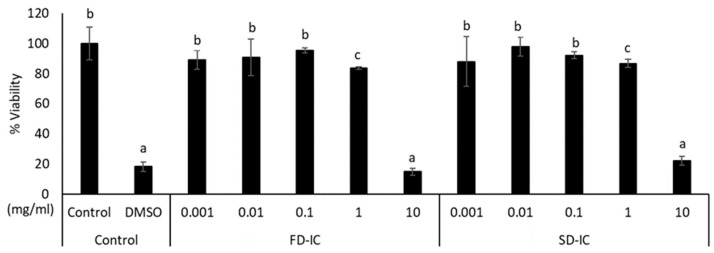
Effect of FD-IC and SD-IC on CCD-18 cell viability. Control refers to untreated cells and DMSO (50%) was used as a death control. Different letters indicate significant differences between samples (Tukey’s test; *p* < 0.05).

Figure 4 shows the effect of FD-IC and SD-IC after IC treatment on CCD-18 cells. As expected, the positive control DMSO reduced the cell viability (about 80% of reduction). The FD-IC and SD-IC treatments did not significantly (*p* > 0.05) reduce viability at any concentration, except at 10 mg/mL. The concentration used for the study of ROS formation in these cells was 0.1 mg/mL, as previously selected for Caco-2 cells.

**Figure 5:**
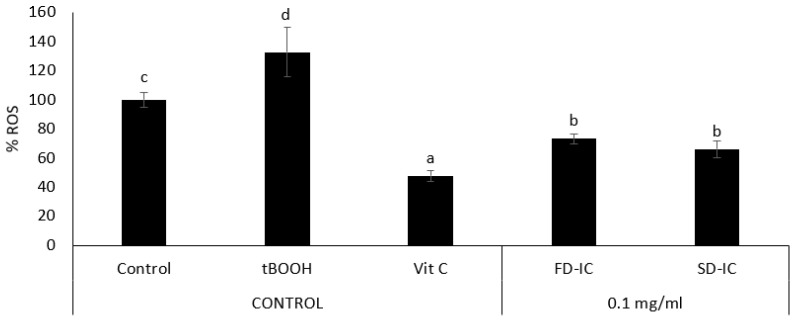
Effect of FD-IC and SD-IC (0.1 mg/mL) on intracellular ROS formation under physiological conditions in Caco-2. Untreated cells were considered as a physiological control, tBOOH (1 mM) was used as an oxidation control, and vitamin C (0.1 mg/mL) was used as an antioxidant control. Different letters indicate significant differences between samples (Tukey’s test; *p* < 0.05).

**Figure 6:**
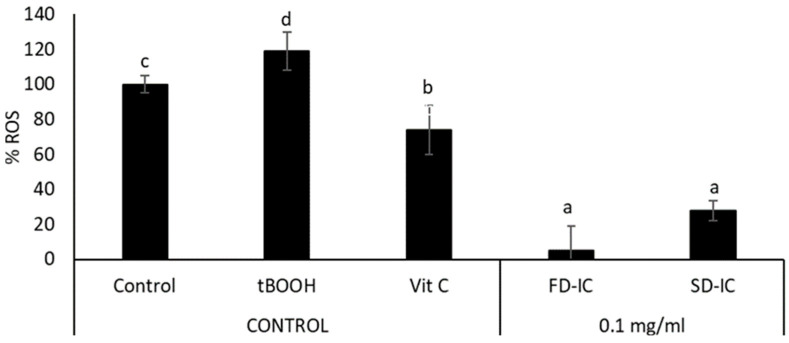
Effect of FD-IC and SD-IC (0.1 mg/mL) on intracellular ROS formation under physiological conditions in CCD-18 cells. Untreated cells were considered as a physiological control, tBOOH (1 mM) was used as an oxidation control, and vitamin C (0.1 mg/mL) was used as an antioxidant control. Different letters indicate significant differences between samples (Tukey’s test; *p* < 0.05).

Figure 5 shows the effect of the FD-IC and SD-IC treatments on the intracellular ROS formation. The studied powders showed a significant reduction (*p* < 0.05) of intracellular ROS formation under physiological conditions. The effect of samples was not significantly different (*p* > 0.05). To the authors’ knowledge, the present study is the first one to assess the effects of IC on ROS formation and the effect of the coffee fruit cascara in human colon cancer cells (Caco-2). The development and progression of colorectal cancer have been linked to oxidative stress, which is also related to inflammation and DNA damage [86]. Both endogenous and exogenous antioxidants can reduce the effects produced by ROS, such as phenolic compounds [87]. The results obtained in cell-based assays indicate that FD-IC and SD-IC could help to decrease tumour progression in colon cancer by reducing intracellular ROS formation. In the case of normal colon cells, both treatments statistically significantly reduced intracellular ROS production, without a statistical difference between them (Figure 6). Oxidative stress causes damage to cellular structures and functions, leading to the development of pathological conditions such as inflammation, cancer, and neurodegenerative diseases [88]; therefore, the maintenance of low ROS levels in normal colon cells could be of interest.

#### 3.3.3. Anti-Inflammatory Properties

**Figure 7:**
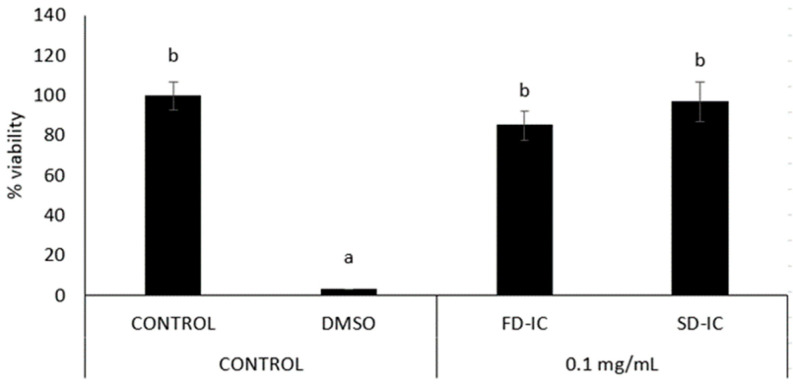
Effect of FD-IC and SD-IC on RAW 264.7 macrophages’ cell viability. Control refers to untreated cells and DMSO (50%) was used as a death control. Different letters indicate significant differences between samples (Tukey’s test; *p* < 0.05).

Before the analysis of anti-inflammatory properties, the effect of samples (0.1 mg/mL) on cell viability was evaluated by the MTT assay. The treatment of the sample with a concentration of 0.1 mg/mL did not affect the viability of RAW 264.7 (Figure 7).

**Figure 8:**
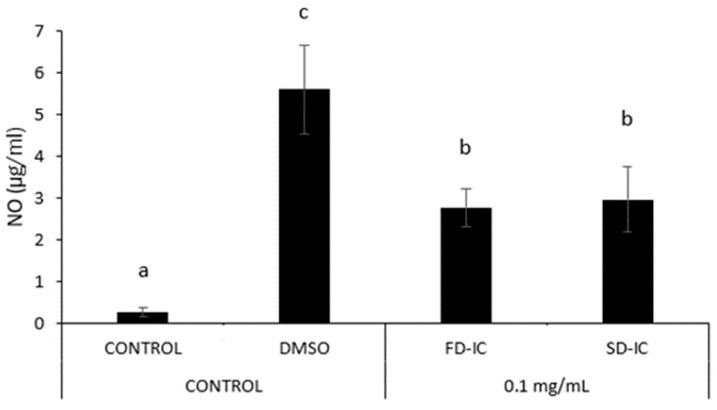
Effect of SD-IC and FD-IC (0.1 mg/mL) on NO formation in RAW 264.7 mouse macrophages. Control refers to untreated cells and LPS (1 µg/mL) was used as a pro-inflammatory control. The pretreatment assay involved treating cells with samples for 24 h, followed by the addition of LPS (1 µg/mL) for another 24 h. In the pretreatment and co-administration assay, cells were treated with samples for 24 h, followed by simultaneous administration of LPS and the sample for another 24 h. The bars indicate the mean values, with error bars representing the standard error of the mean (SEM). Different letters represent significant differences (Tukey’s test; *p* < 0.05).

Figure 8 shows NO formation in RAW 264.7 macrophages induced by LPS, and the co-administration of instant beverages obtained through two dehydration processes. A significant reduction (*p* < 0.05) in NO formation was observed in the two samples under study. The values obtained with the co-administration of the two samples were not significantly different (*p* > 0.05), suggesting that their anti-inflammatory potential was similarly affected by the two dehydration processes.

The anti-inflammatory effect of coffee fruit cascara may be due to the methylxanthines and phenolic compounds present, especially caffeine and chlorogenic and caffeic acid [10,89,90]. Caffeic and chlorogenic acid, as well as caffeine, have shown anti-inflammatory potential in LPS-stimulated RAW 264.7 macrophages [91,92]. In general, the content of phenolic compounds decreases with increasing temperature [93]. Budryn et al. [94] described that a temperature above 203 °C produced a high level of degradation of caffeoylquinic acids, but at 190°, this degradation was lower (24%), such as for chlorogenic acid. However, chlorogenic and caffeic acid are less thermally resistant than caffeine [93]. Based on these data, it could be suggested that at the temperatures used in the processing of the samples, the degradation of the different phenolic compounds present would be low.

Gallego-Barcelo et al. [95] studied the effects of the same product (IC) at 10 mg/mL on the general health and brain–gut axis parameters of healthy male and female rats. Their findings indicate that a regular intake of IC did not induce significant alterations in body weight, gastrointestinal motility, or behavioural parameters associated with anxiety or anhedonia. Notably, the differences observed between sexes were consistent with known sex dimorphism in rodent physiology, with males exhibiting higher food intake and slight morphometric changes in gastrointestinal organs compared to females. Importantly, neither males nor females exhibited signs of toxicity or abnormal weight modifications after consuming IC. These results suggest that IC consumption at the studied concentration does not adversely affect the brain–gut axis in healthy rats and supports the safety of IC as a beverage option.

### 3.4. Environmental Impact Quantification

To compare the drying processes in terms of environmental performance, their impacts were quantified. In this sense, the chosen methodology for calculation was the most developed: life cycle assessment (LCA). This methodology is standardized based on ISO regulation (ISO14040 and ISO14044) and provides quantitative data on different categories of environmental impacts of the processes. In fact, it can provide a comprehensive view of the performance of the processes to improve them.

**Figure 9:**
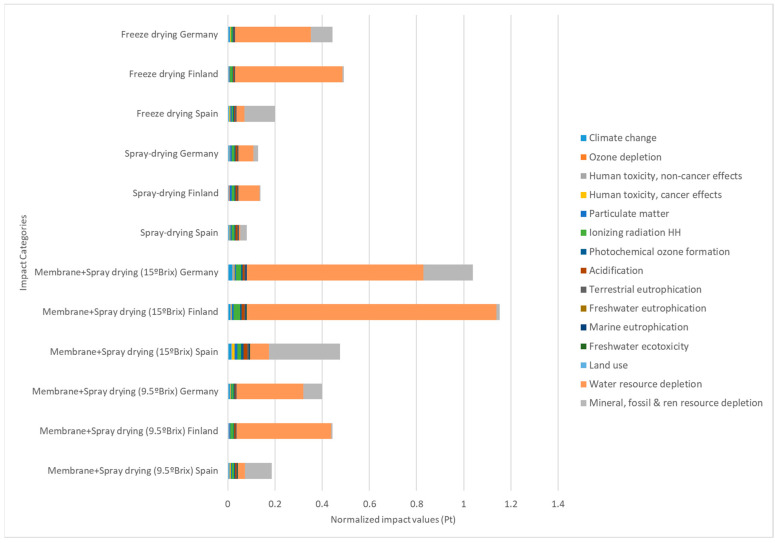
Impact assessment comparison of 1 kg of dried instant cascara (functional unit) by freeze drying and spray drying. Impact values are shown in normalized points (Pts) to standardize their values as specified in the ILCD calculation method.

The comparison in terms of the environmental impacts of the drying methods applied to the production of dried instant cascara beverages can be seen in Figure 9. The analysis was performed using 1 kg of dried cascara as a functional unit. The aim of using LCA in this article was a comparison of the processes. Therefore, the values are not significant by themselves; nevertheless, they represent a contrast. For this reason, the values shown in Figure 9 are expressed as normalized values, called normalized impact points (Pts) by the software SimaPro Software (version 8.2.3). The different impact categories included in the LCA calculation can be seen in detail in Figure 9. Moreover, the same study was carried out considering three scenarios; i.e., the same processes were carried out in three countries (Spain, Germany, and Finland) with different energy mixes. In this sense, the total impact of the FD process is more than double that of the impacts produced by SD when comparing within each country. The impact is high in the combined process of membrane filtration and spray drying (concentrated spray drying), independently of the concentration in °Brix reached by the membrane filtration. These results could be associated with the energy consumption of all the processes. These increased environmental impacts are notable in all impact categories, for example for the carbon footprint, which in this LCA calculation method is called “climate change”; the neat values expressed in kg CO_2_ equivalent per functional unit (1 kg IC) in the case of Spain were 750 kg eqCO_2_ for SD and 720 kg eqCO_2_ for FD, while the combined method varied from 789 kg eqCO_2_ concentrating up to 9.5 °Brix to 1830 kg eqCO_2_. As said before, these impacts are associated with energy consumption; in the present study, the Spanish energy mix was used as electrical power. But by performing the same calculation using Germany’s or Finland’s energy mix, the neat values were increased.

Several scientific articles applied the LCA methodology to quantify several aspects related to coffee; for example, it has been used to analyse the cultivation and initial stages [96], the decaffeination process [97], the supply chain of coffee [98], and the preparation of coffee [99]. Moreover, LCA has been applied, of course, in coffee by-product management, where the main product is spent coffee grounds [100], but also to other by-products, such as coffee silverskin [101]. Nevertheless, this is the first approach to studying the environmental impacts associated with coffee fruit cascara production.

### 3.5. A Business Model and an Operations and Investment Plan

Coffee by-products that are produced during the process of obtaining green coffee beans are primarily generated in the countries where the coffee is grown. However, drying the ripe pulp (cascara) under controlling and standardized conditions in these countries and exporting it to Europe can be an excellent way to use it as a traditional ingredient in developing new foods without incurring high additional costs. The data presented in the present paper support the feasibility of this practice, which can have a tremendous socioeconomical and environmental impact.

The technology readiness level (TRL) is an accepted way of measuring the degree of maturity of an R&D technology. There are nine levels ranging from the lowest or basic idea level to the last level, 9, representing a system successfully tested in a real environment.

The first three levels (TRL1–TRL3, chasm I) correspond to the most basic technological research until reaching a first proof of concept; translated to R&D, this would be the research phase or laboratory environment. Technological development progresses in the intermediate levels (TRL4–TRL7, chasm II) until reaching a prototype or non-marketable demonstration and, finally, its market launch and deployment (TRL8–TRL9, chasm III) [102].

Therefore, according to the Ministerio de Industria [102], from a testing and validation point of view, the classification by level would be as follows. TRL 1: basic idea; TRL 2: formulated concept or technology; TRL 3: proof of concept; TRL 4: component-level validation in a laboratory; TRL 5: component-level validation in a relevant environment (with conditions that closely approximate or closely simulate conditions in a real environment); TRL 6: system or subsystem validation in a relevant environment; TRL 7: system validation in a real environment; TRL 8: full validation and certification in a real environment; and TRL 9: successful testing in a real environment.

For the product developed in this work, laboratory tests have already been performed. So, there is a product that is already developed, formulated, and tested for its properties. The product is currently between levels 5 and 6 on the modified technology readiness level (mTRL) scale.

Moreover, there are key partners such as suppliers of raw materials, suppliers of auxiliary materials, and investors who are interested in the development of instant cascara. For this, there are some key activities that have already been implemented, such as product reformulation; some key future activities such as the presentation of the reformulated product through a consumer panel or the dissemination of the product to allow analysis of big consumer data, showing the acceptability of the product; and further, a possible reformulation towards the tastes of consumers in aspects such as appearance, colour, smell, taste, and acceptability. The preliminary analysis of the preferences of consumers performed in the present study supports their purchase intent and the product market potential of the dried ripe coffee cherry pulp soluble powder.

According to Iriondo-DeHond et al. [10], 90% of the edible parts of the coffee cherry are lost from seed to cup. Giving food waste a second life is key to achieving a sustainable food system. So, the coffee industry is exploring circular economy practices to drive the long-term shift from the traditional “take–make–dispose” approach, which is an unsustainable model [103], towards utilizing the by-products of the coffee process.

Considering that the coffee industry discards 96% of the coffee fruit, generating 3 kg of waste per person per year, the added value of instant coffee cascara is to reduce this waste. Currently, these residues are mainly used to produce biofuels. However, giving them a second life would reduce pollution and fight against food waste by reusing a by-product of the coffee industry for human consumption [104], in line with the Sustainable Development Goals from the United Nations for a more sustainable food system and framed within the circular economy.

In the first place, companies that produce products with coffee fruit cascara have entered the market with force and are becoming more and more numerous. Products such as coffee tea, cascara sparkling drinks, chocolates, coffee cascara flours, or yogurts infused with cascara have been commercially available in Europe since the approval of the use of the cascara as a food at the beginning of 2022.

As mentioned above, the by-products of the coffee industry are generally produced in the countries of origin of the coffee. However, the waste could be exported to Europe, or this idea could be implemented in the origin countries to give it a second life. If Spain were to import the coffee fruit cascara of all the coffee it consumes, it would have a Total Addressable Market (TAM) of 2.5 million tonnes of cascara. However, not everything is part of the Serviceable Available Market (SAM). Focusing on the Community of Madrid, where the R&D centre is located, the available market would be 375 thousand tonnes of cascara. Finally, considering the production capacity of the key partners, it is believed that reaching 1% of this market would be a good starting point.

To assess the readiness of the instant cascara beverage, the product is currently between levels 5 and 6 on the modified technology readiness level (mTRL) scale. A prototype of the product has been realised and a proto-qualification has been performed with customers. The food seems to have crossed chasm I and is heading towards chasm II. Although it is not clear what time scale is required for this new food to move from chasm I to chasm II, it seems clear that this duration will be influenced mainly by external and compound factors, such as the definition of market spaces, distribution, strategic positioning, and the business model, among other factors.

Instant beverages from dried ripe coffee cherry pulp (which are traditional in third-world countries) with cheap raw materials constitute “a more sustainable alternative and a values-based production chain that values people, planet, and profit equally” [103]. Fulfilling corporate social responsibility objectives can help companies create a competitive and unique beverage product. A prototype of the new product in a pilot plant has been developed. Data on small-scale consumer panels supported the market potential of dried ripe coffee cherry pulp soluble powder, also called instant cascara. The following steps in this process will be to conduct a small-scale consumer panel to evaluate the market potential of the reformulated product, offer the instant cascara in a real environment, such as a food fair or supermarket shelves, and finally develop the product at an industrial scale. Once all the necessary steps have been completed, the product will be ready to be released on the market.

## 4. Conclusions

Spray drying can be used to produce soluble powders from dried ripe coffee cherry pulp, with antioxidant and anti-inflammatory properties with a lower environmental impact than freeze drying due to its lower energy consumption. In addition, the product obtained is beneficial for consumers’ health due to its concentration of nutrients and bioactive compounds at a dose of 10 mg/mL. Here, phenolic compounds and methylxanthines were detected in the digested fraction of the beverage. The LCA method calculated the impact of the process in terms of “climate change”, resulting in values expressed in kg CO_2_ equivalent per functional unit (1 kg of IC). In the case of Spain, the impact was estimated to be 750 kg eqCO_2_ for spray-dried and 720 kg eqCO_2_ for freeze-dried. Spray drying allows the production of a much more environmentally friendly product. The product is currently between levels 5 and 6 on the modified technology readiness level (mTRL) scale.

## Figures and Tables

**Table 1:** Sensory acceptance of IC beverages using a nine-point hedonic scale.

Attributes	Adults	Adolescents
Tabifruit		
Overall liking	5.12 ± 1.81	4.92 ± 2.31 ^b^
Visual appearance	6.12 ± 2.29	4.94 ± 2.69
Smell	5.00 ± 2.20	4.40 ± 2.67
Taste	4.23 ± 2.17	3.45 ± 2.16
IC 4 mg/mL		
Overall liking	5.23 ± 1.84	5.00 ± 1.99 ^b^
Visual appearance	5.88 ± 2.49	4.73 ± 2.59
Smell	4.73 ± 2.17	4.48 ± 7.75
Taste	4.38 ± 2.18	3.61 ± 2.41
IC 10 mg/mL		
Overall liking	5.16 ± 2.08	3.95 ± 1.98 ^a^
Visual appearance	6.08 ± 2.42	4.52 ± 2.47
Smell	4.88 ± 2.43	4.47 ± 2.64
Taste	4.36 ± 2.41	2.71 ± 2.06

Data are expressed as the means ± standard deviation. Values in each column with different letters differ significantly when comparing the same attribute in the three products (Tukey test, *p* < 0.05).

**Table 2:** Physical parameters of dried ripe coffee cherry pulp soluble powders obtained by freeze drying and spray drying (appearance, moisture, and colour) and their beverages at 10 mg/mL (appearance, pH, °Brix, colour, and UV-Vis).

Parameters	Freeze Drying		Spray Drying		*p* < 0.05
Appearance	FD-IC 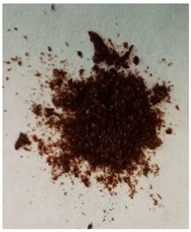	FD-IC beverage 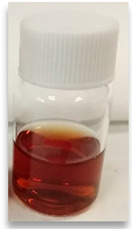	SD-IC 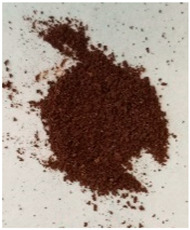	SD-IC beverage 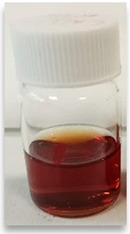	
Moisture (%)	5.32 ± 0.31		3.71 ± 0.34		*
pH	3.44 ± 0.11		4.31 ± 0.04		*
°Brix	1		1		
Colour (lightness parameter)	FD-IC 85.89 ± 0.49	FD-IC beverage 144.73 ± 0.29	SD-IC 90.92 ± 0.77	SD-IC beverage 102.28 ± 1.07	*
UV-VIS	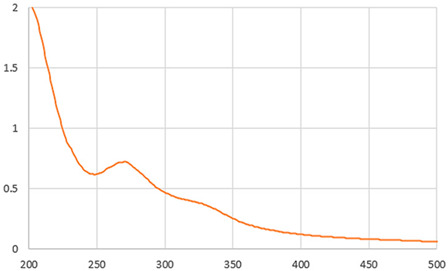		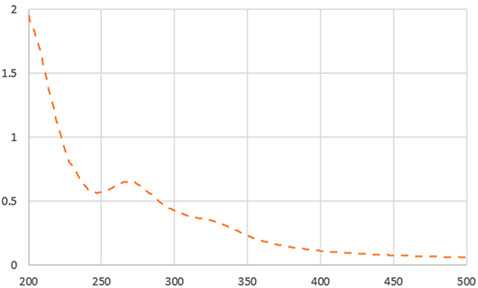		

Data are expressed as the means ± standard deviation. Asterisks indicate a significant difference for both samples studied (*t*-Student test, *p* < 0.05).

**Table 3:** Phytochemical composition (methylxanthines and phenolic compounds) of IC powders.

Compounds Studied	Freeze Drying	Spray Drying	*p* < 0.05
**Methylxanthines**			
Caffeine (mg/g of dried sample)	12.33 ± 2.43	35.47 ± 0.44	*
Theobromine (µg/g of dried sample)	72.20 ± 4.79	31.72 ± 3.49	*
Theophylline (µg/g of dried sample)	117.84 ± 1.25	25.47 ± 2.81	*
Total (mg/g of dried sample)	12.52	35.52	*
**Phenolic compounds**			
Isoflavones			
Epicatechin (µg/g of dried sample)	1.32 ± 0.22	3.08 ± 0.33	*
Mangiferin (µg/g of dried sample)	176.55 ± 29.24	662.84 ± 38.83	*
Rutin (µg/g of dried sample)	88.14 ± 19.80	132.20 ± 2.19	
Total (mg/g of dried sample)	0.26	0.79	*
Hydroxycinnamic acids			
p-coumaroylquinic acid (µg/g of dried sample)	242.45 ± 45.82	162.43 ± 5.48	*
Caffeoylquinic acid (µg/g of dried sample)	890.41 ± 284.47	1191.70 ± 448.53	
3-O-caffeoylquinic acid (chlorogenic acid) (µg/g of dried sample)	2851.58 ± 526.78	2986.58 ± 54.55	
4-O-feruloylquinic acid (µg/g of dried sample)	21.81 ± 2.12	16.44 ± 4.76	
5-O-feruloylquinic acid (µg/g of dried sample)	350.08 ± 70.27	478.13 ± 14.33	*
3,4-di-O-caffeoylquinic acid (µg/g of dried sample)	69.67 ± 17.93	236.98 ± 62.79	
4,5-di-O-caffeoylquinic acid (µg/g of dried sample)	32.54 ± 4.40	88.84 ± 6.43	*
Total (mg/g of dried sample)	4.45	5.16	
Anthocyanins			
Cyanidin-3-O-glucoside (µg/g of dried sample)	1.32 ± 0.23	N.D.	
Cyanidin-3-O-rutinoside (µg/g of dried sample)	5.25 ± 1.40	N.D.	
Total (mg/g of dried sample)	0.0065	N.D.	
TOTAL (mg/g of dried sample)	17.23	41.47	*

Data are expressed as the means ± standard deviation. N.D., not detected. Asterisks indicate a significant difference for both samples studied (*t*-Student test, *p* < 0.05).

**Table 4:** Phytochemical composition (methylxanthines and phenolic compounds) of digests of beverages prepared with the powdered extracts at a concentration of 10 mg/mL.

Compounds Studied (µg eq./mL of Digest)	FD-IC Digested	SD-IC Digested	*p* < 0.05
**Methylxanthines**			
Caffeine	867.55 ± 5.12	663.27 ± 3.21	*
Theobromine	1.76 ± 0.04	1.76 ± 0.06	
Theophylline	0.91 ± 0.05	1.02 ± 0.06	
Total	870.22	666.05	*
**Phenolic compounds**			
Isoflavones			
Catechin hexoside	0.35 ± 0.01	0.44 ± 0.03	*
Mangiferin	4.52 ± 0.31	4.68 ± 0.44	
Rutin	0.25 ± 0.02	0.23 ± 0.04	
Total	5.12	5.35	
Hydroxycinnamic acids			
p-coumaroylquinic acid	0.80 ± 0.03	1.01 ± 0.07	*
Caffeoylquinic acid	23.38 ± 11.69	19.12 ± 9.56	
3-O-caffeoylquinic acid (chlorogenic acid)	23.08 ± 1.83	27.88 ± 2.27	*
4-O-feruloylquinic acid	1.24 ± 0.10	0.85 ± 0.07	*
5-O-feruloylquinic acid	4.78 ± 0.38	4.88 ± 0.47	
3,4-di-O-caffeoylquinic acid	0.61 ± 0.30	0.45 ± 0.23	
4,5-di-O-caffeoylquinic acid	0.56 ± 0.06	0.37 ± 0.04	*
Total	54.45	54.56	
TOTAL	929.79	725.96	*

Data are expressed as the means ± standard deviation. * indicate a significant difference for both samples studied (*t*-Student test, *p* < 0.05).

**Table 5:** Antioxidant capacity of instant cascara obtained by freeze drying and spray drying, respectively, analysed in terms of total phenolic content (TPC), DPPH, and ABTS. Data are expressed as mg eq. of CGA/g of dried sample. Values are means ± standard deviation.

Assay	Freeze Drying	Spray Drying
TPC	92.64 ± 6.75	99.56 ± 3.45
DPPH	62.29 ± 1.60	78.56 ± 29.18
ABTS	242.92 ± 8.66	249.25 ± 7.06

Data are expressed as the means ± standard deviation. No significant differences were found for both samples studied (*t*-Student test, *p* < 0.05).

**Table 6:** Antioxidant capacity of digested extracts analysed by ABTS.

Assay (mmol eq. CGA/l of Digest)	Freeze Drying	Spray Drying	*p* < 0.05
ABTS	24.2 ± 1.83	30.59 ± 1.89	*

Data are expressed as the means ± standard deviation. * indicates a significant difference for both samples studied (*t*-Student test, *p* < 0.05).

## Data Availability

The original contributions presented in the study are included in the article, further inquiries can be directed to the corresponding author.

## References

[1] EFSA (2021). Technical Report on the Notification of Dried Cherry Pulp from *Coffea arabica* L. and *Coffea canephora* Pierre Ex A. Froehner as a Traditional Food from a Third Country Pursuant to Article 14 of Regulation (EU) 2015/2283. EFSA Support. Publ..

[2] Commission Implementing Regulation (EU) (2022). 2022/47 of 13 January 2022 Authorising the Placing on the Market of *Coffea arabica* L. and/or *Coffea canephora* Pierre Ex A. Froehner Dried Cherry Pulp and Its Infusion as a Traditional Food from a Third Country under Regulation (EU) 2015/2283 of the European Parliament and of the Council and Amending Commission Implementing Regulation (EU) 2017/2470.

[3] Muzaifa M., Rahmi F. (2021). Syarifudin Utilization of Coffee By-Products as Profitable Foods-A Mini Review. IOP Conf. Ser. Earth Environ. Sci..

[4] Nestle NESCAFÉ NATIV Cascara. https://www.nestle.com.au/en/brands/cascara.

[5] TEAGLEE Cascara Tea Blends. https://www.teaglee.com/collections.

[6] Sales A.L., Iriondo-DeHond A., DePaula J., Ribeiro M., Ferreira I.M.P.L.V.O., Miguel M.A.L., del Castillo M.D., Farah A. (2023). Intracellular Antioxidant and Anti-Inflammatory Effects and Bioactive Profiles of Coffee Cascara and Black Tea Kombucha Beverages. Foods.

[7] Bella Vista Coffee KaldiKombucha. https://kaldikombucha.com/.

[8] Supracafe Tabifruit. https://www.supracafe.com/blogs/news/conoces-la-infusion-de-cascara-de-cafe-supracafe-maximiza-su-sostenibilidad-con-tabifruit.

[9] Coffeeberry^®^ Cascara is a Patented Extract Derived from Upcycled Coffee Fruit. https://www.futureceuticals.com/coffeeberry-cascara.

[10] Iriondo-DeHond A., Elizondo A.S., Iriondo-DeHond M., Ríos M.B., Mufari R., Mendiola J.A., Ibañez E., del Castillo M.D. (2020). Assessment of Healthy and Harmful Maillard Reaction Products in a Novel Coffee Cascara Beverage: Melanoidins and Acrylamide. Foods.

[11] Sharma A., Jana A.H., Chavan R.S. (2012). Functionality of Milk Powders and Milk-Based Powders for End Use Applications—A Review. Compr. Rev. Food Sci. Food Saf..

[12] Dutta S., Moses J.A., Anandharamakrishnan C. (2018). Modern Frontiers and Applications of Spray-Freeze-Drying in Design of Food and Biological Supplements. J. Food Process Eng..

[13] Ghirişan A., Miclăuş V. (2017). Comparative Study of Spray-Drying and Freeze-Drying on the Soluble Coffee Properties. Stud. Univ. Babes-Bolyai Chem..

[14] Ballesteros L.F., Ramirez M.J., Orrego C.E., Teixeira J.A., Mussatto S.I. (2017). Encapsulation of Antioxidant Phenolic Compounds Extracted from Spent Coffee Grounds by Freeze-Drying and Spray-Drying Using Different Coating Materials. Food Chem..

[15] Ishwarya S.P., Anandharamakrishnan C. (2015). Spray-Freeze-Drying Approach for Soluble Coffee Processing and Its Effect on Quality Characteristics. J. Food Eng..

[16] Darniadi S., Ho P., Murray B.S. (2018). Comparison of Blueberry Powder Produced via Foam-Mat Freeze-Drying versus Spray-Drying: Evaluation of Foam and Powder Properties. J. Sci. Food Agric..

[17] Sreevalsan S., Safe S. (2013). Reactive Oxygen Species and Colorectal Cancer. Curr. Color. Cancer Rep..

[18] Zuo L., Prather E.R., Stetskiv M., Garrison D.E., Meade J.R., Peace T.I., Zhou T. (2019). Inflammaging and Oxidative Stress in Human Diseases: From Molecular Mechanisms to Novel Treatments. Int. J. Mol. Sci..

[19] Tontul I., Topuz A. (2017). Spray-Drying of Fruit and Vegetable Juices: Effect of Drying Conditions on the Product Yield and Physical Properties. Trends Food Sci. Technol..

[20] Fontes C.P.M.L., Silva J.L.A., Sampaio-Neta N.A., da Costa J.M.C., Rodrigues S. (2014). Dehydration of Prebiotic Fruit Drinks by Spray Drying: Operating Conditions and Powder Characterization. Food Bioprocess Technol..

[21] Penner M., Nielsen S. (2017). Ultraviolet, Visible, and Fluorescence Spectroscopy. Food Analysis. Food Science Text Series.

[22] Iriondo-DeHond A. (2020). Assessment of Healthy and Harmful Maillard Reaction Products in a Novel Co Ff Ee Cascara Beverage. Foods.

[23] Contini M., Baccelloni S., Massantini R., Anelli G. (2008). Extraction of Natural Antioxidants from Hazelnut (*Corylus avellana* L.) Shell and Skin Wastes by Long Maceration at Room Temperature. Food Chem..

[24] Hollebeeck S., Borlon F., Schneider Y.J., Larondelle Y., Rogez H. (2013). Development of a Standardised Human in Vitro Digestion Protocol Based on Macronutrient Digestion Using Response Surface Methodology. Food Chem..

[25] Martinez-Saez N., Hochkogler C.M., Somoza V., del Castillo M.D. (2017). Biscuits with No Added Sugar Containing Stevia, Coffee Fibre and Fructooligosaccharides Modifies α-Glucosidase Activity and the Release of GLP-1 from HuTu-80 Cells and Serotonin from Caco-2 Cells after In Vitro Digestion. Nutrients.

[26] Re R., Pellegrini N., Proteggente A., Pannala A., Yang M., Rice-Evans C. (1999). Antioxidant Activity Applying an Improved ABTS Radical Cation Decolorization Assay. Free Radic. Biol. Med..

[27] Islam M.K., Sostaric T., Lim L.Y., Hammer K., Locher C. (2020). Development and Validation of an HPTLC–DPPH Assay and Its Application to the Analysis of Honey. J. Planar Chromatogr. Mod. TLC.

[28] Aljadi A.M., Kamaruddin M.Y. (2004). Evaluation of the Phenolic Contents and Antioxidant Capacities of Two Malaysian Floral Honeys. Food Chem..

[29] Bakondi E., Gönczi M., Szabó É., Bai P., Pacher P., Gergely P., Kovács L., Hunyadi J., Szabó C., Csernoch L. (2003). Role of Intracellular Calcium Mobilization and Cell-Density-Dependent Signaling in Oxidative-Stress-Induced Cytotoxicity in HaCaT Keratinocytes. J. Investig. Dermatol..

[30] Bedoya-Ramírez D., Cilla A., Contreras-Calderón J., Alegría-Torán A. (2017). Evaluation of the Antioxidant Capacity, Furan Compounds and Cytoprotective/Cytotoxic Effects upon Caco-2 Cells of Commercial Colombian Coffee. Food Chem..

[31] Iriondo-DeHond A., Ramírez B., Escobar F.V., del Castillo M.D. (2019). Antioxidant Properties of High Molecular Weight Compounds from Coffee Roasting and Brewing Byproducts. Bioact. Compd. Health Dis..

[32] Benayad Z., Martinez-Villaluenga C., Frias J., Gomez-Cordoves C., Es-Safi N.E. (2014). Phenolic Composition, Antioxidant and Anti-Inflammatory Activities of Extracts from Moroccan Opuntia Ficus-Indica Flowers Obtained by Different Extraction Methods. Ind. Crops Prod..

[33] Hauschild M., Goedkoop M., Guinee J., Heijungs R., Huijbregts M., Jolliet O., Margni M., De Schryver A., Pennington D., Pant R. (2011). Recommendations for Life Cycle Impact Assessment in the European Context—Based on Existing Environmental Impact Assessment Models and Factors (International Reference Life Cycle Data System—ILCD Handbook).

[34] Nielsen C., Lund M. (2014). A Brief History of the Business Model Concept. The Basics of Business Models.

[35] Bocken N.M.P., Rana P., Short S.W. (2015). Value Mapping for Sustainable Business Thinking. J. Ind. Prod. Eng..

[36] Iriondo-DeHond A. (2019). Validation of Coffee By-Products as Food Ingredients for a Sustainable Nutrition and Health. Ph.D. Thesis.

[37] Schuck P., Jeantet R., Bhandari B., Chen X.D., Perrone Í.T., de Carvalho A.F., Fenelon M., Kelly P. (2016). Recent Advances in Spray Drying Relevant to the Dairy Industry: A Comprehensive Critical Review. Dry. Technol..

[38] Pereira D.C.d.S., Beres C., Gomes F.d.S., Tonon R.V., Cabral L.M.C. (2020). Spray Drying of Juçara Pulp Aiming to Obtain a “Pure” Powdered Pulp without Using Carrier Agents. Dry. Technol..

[39] Sobulska M., Zbicinski I. (2021). Advances in Spray Drying of Sugar-Rich Products. Dry. Technol..

[40] Tantiyani N., Othman A., Elmi M., Mohd F. (2019). Drying of Instant Coffee in a Spray Dryer. J. Kejuruter..

[41] Mennella J.A. (2014). Ontogeny of Taste Preferences: Basic Biology and Implications for Health1-5. Am. J. Clin. Nutr..

[42] Wilkowska A., Ambroziak W., Czyzowska A., Adamiec J. (2016). Effect of Microencapsulation by Spray-Drying and Freeze-Drying Technique on the Antioxidant Properties of Blueberry (*Vaccinium myrtillus*) Juice Polyphenolic Compounds. Pol. J. Food Nutr. Sci..

[43] Nicoli M.C., Calligaris S., Manzocco L. (2009). Shelf-Life Testing of Coffee and Related Products: Uncertainties, Pitfalls, and Perspectives. Food Eng. Rev..

[44] Forsido S.F., Welelaw E., Belachew T., Hensel O. (2021). Effects of Storage Temperature and Packaging Material on Physico-Chemical, Microbial and Sensory Properties and Shelf Life of Extruded Composite Baby Food Flour. Heliyon.

[45] Quek S.Y., Chok N.K., Swedlund P. (2007). The Physicochemical Properties of Spray-Dried Watermelon Powders. Chem. Eng. Process. Process Intensif..

[46] Tonon R.V., Brabet C., Hubinger M.D. (2008). Influence of Process Conditions on the Physicochemical Properties of Açai (*Euterpe oleraceae* Mart.) Powder Produced by Spray Drying. J. Food Eng..

[47] Ferrari C.C., Germer S.P.M., de Aguirre J.M. (2012). Effects of Spray-Drying Conditions on the Physicochemical Properties of Blackberry Powder. Dry. Technol..

[48] Abdullah Z., Taip F.S., Kamal S.M.M., Abdul Rahman R.Z. (2020). Nonlinear Model-Based Inferential Control of Moisture Content of Spray Dried Coconut Milk. Foods.

[49] Maharani S., Mustikawati I., Nailufhar L., Istiqomah S. (2021). The Effect of Brewing Time on PH Values, Polyphenols Content, and Antioxidant Activities of Coffee Husk Tea (Cascara Tea). J. Phys. Conf. Ser..

[50] Friedman M., Jürgens H.S. (2000). Effect of PH on the Stability of Plant Phenolic Compounds. J. Agric. Food Chem..

[51] Narita Y., Inouye K. (2013). Degradation Kinetics of Chlorogenic Acid at Various PH Values and Effects of Ascorbic Acid and Epigallocatechin Gallate on Its Stability under Alkaline Conditions. J. Agric. Food Chem..

[52] Saarniit K., Lang H., Kuldjärv R., Laaksonen O., Rosenvald S. (2023). The Stability of Phenolic Compounds in Fruit, Berry, and Vegetable Purees Based on Accelerated Shelf-Life Testing Methodology. Foods.

[53] Deotale S.M., Dutta S., Moses J.A., Anandharamakrishnan C. (2022). Influence of Drying Techniques on Sensory Profile and Chlorogenic Acid Content of Instant Coffee Powders. Meas. Food.

[54] Pua A., Choo W.X.D., Goh R.M.V., Liu S.Q., Cornuz M., Ee K.H., Sun J., Lassabliere B., Yu B. (2021). A Systematic Study of Key Odourants, Non-Volatile Compounds, and Antioxidant Capacity of Cascara (Dried Coffea Arabica Pulp). LWT.

[55] Christensen B.E., Strand S.P., Basset C., Kristiansen K.A., Ulset A.S.T., Ballance S., Granum P.E. (2018). Macromolecular Acidic Coating Increases Shelf Life by Inhibition of Bacterial Growth. Int. J. Food Microbiol..

[56] Murlida E., Noviasari S., Nilda C., Rohaya S., Rahmi F., Muzaifa M. (2021). Chemical Characteristics of Cascara Tea from Several Varieties of Coffee in Aceh Province. IOP Conf. Ser. Earth Environ. Sci..

[57] Rohaya S., Anwar S.H., Amhar A.B., Sutriana A., Muzaifa M. (2023). Antioxidant Activity and Physicochemical Composition of Coffee Pulp Obtained from Three Coffee Varieties in Aceh, Indonesia. IOP Conf. Ser. Earth Environ. Sci..

[58] Frizon C.N.T., Nisgoski S. (2020). Color Parameters to Predict Moisture and Tannin Content in Yerba Mate Process. Floresta e Ambiente.

[59] Dey P., Kundu A., Kumar A., Gupta M., Lee B.M., Bhakta T., Dash S., Kim H.S. (2020). Analysis of Alkaloids (Indole Alkaloids, Isoquinoline Alkaloids, Tropane Alkaloids).

[60] Monteiro J.P., Alves M.G., Oliveira P.F., Silva B.M. (2016). Structure-Bioactivity Relationships of Methylxanthines: Trying to Make Sense of All the Promises and the Drawbacks. Molecules.

[61] Jeszka-Skowron M., Frankowski R., Zgoła-Grześkowiak A. (2020). Comparison of Methylxantines, Trigonelline, Nicotinic Acid and Nicotinamide Contents in Brews of Green and Processed Arabica and Robusta Coffee Beans—Influence of Steaming, Decaffeination and Roasting Processes on Coffee Beans. LWT.

[62] Touil A., Peczalski R., Timoumi S., Zagrouba F. (2013). Influence of Air Temperature and Humidity on Dehydration Equilibria and Kinetics of Theophylline. J. Pharm..

[63] Jokić S., Gagić T., Knez E., Ubarić D., Kerget M. (2018). Separation of Active Compounds from Food By-Product (Cocoa Shell) Using Subcritical Water Extraction. Molecules.

[64] Mohdaly A.A.A., Roby M.H.H., Sultan S.A.R., Groß E., Smetanska I. (2022). Potential of Low Cost Agro-Industrial Wastes as a Natural Antioxidant on Carcinogenic Acrylamide Formation in Potato Fried Chips. Molecules.

[65] Lin D., Xiao M., Zhao J., Li Z., Xing B., Li X., Kong M., Li L., Zhang Q., Liu Y. (2016). An Overview of Plant Phenolic Compounds and Their Importance in Human Nutrition and Management of Type 2 Diabetes. Molecules.

[66] Saha S., Sadhukhan P., Sil P.C. (2016). Mangiferin: A Xanthonoid with Multipotent Anti-Inflammatory Potential. BioFactors.

[67] Klingel T., Kremer J.I., Gottstein V., De Rezende T.R., Schwarz S., Lachenmeier D.W. (2020). A Review of Coffee By-Products Including Leaf, Flower, Cherry, Husk, Silver Skin, and Spent Grounds as Novel Foods within the European Union. Foods.

[68] Michalska A., Wojdyło A., Majerska J., Lech K., Brzezowska J. (2019). Qualitative and Quantitative Evaluation of Heat-Induced Changes in Polyphenols and Antioxidant Capacity in *Prunus domestica* L. By-Products. Molecules.

[69] Prata E.R.B.A., Oliveira L.S. (2007). Fresh Coffee Husks as Potential Sources of Anthocyanins. LWT.

[70] Nur Fitriani U.A., Yusuf M., Ilyas F.S. (2021). Spray Drying of Rosella (*Hibiscus sabdariffa* L.) Powder: Effect of Shelf Life on Physicochemical Properties and Cyanidin 3-O-Glucoside. IOP Conf. Ser. Earth Environ. Sci..

[71] Soares M.J., Sampaio G.R., Guizellini G.M., Figueira M.S., Pinaffi A.C.d.C., Soares Freitas R.A.M., Shahidi F., de Camargo A.C., Torres E.A.F.d.S. (2021). Regular and Decaffeinated Espresso Coffee Capsules: Unravelling the Bioaccessibility of Phenolic Compounds and Their Antioxidant Properties in Milk Model System upon in Vitro Digestion. LWT.

[72] Iriondo-Dehond A., Uranga J.A., Del Castillo M.D., Abalo R. (2021). Effects of Coffee and Its Components on the Gastrointestinal Tract and the Brain–Gut Axis. Nutrients.

[73] Machado F., Coimbra M.A., del Castillo M.D., Coreta-Gomes F. (2023). Mechanisms of Action of Coffee Bioactive Compounds—A Key to Unveil the Coffee Paradox. Crit. Rev. Food Sci. Nutr..

[74] Depaula J., Farah A. (2019). Caffeine Consumption through Coffee: Content in the Beverage, Metabolism, Health Benefits and Risks. Beverages.

[75] Graham T.E. (2001). Caffeine and Exercise: Metabolism, Endurance and Performance. Cafeine et Exercice: Metabolisme, Endurance et Performance. Sports Med..

[76] Turck D., Bohn T., Castenmiller J., De Henauw S., Hirsch-Ernst K.I., Maciuk A., Mangelsdorf I., McArdle H.J., Naska A., Pelaez C. (2022). Safety of Dried Coffee Husk (Cascara) from *Coffea arabica* L. as a Novel Food Pursuant to Regulation (EU) 2015/2283. EFSA J..

[77] Esquivel P., Viñas M., Steingass C.B., Gruschwitz M., Guevara E., Carle R., Schweiggert R.M., Jiménez V.M. (2020). Coffee (*Coffea arabica* L.) by-Products as a Source of Carotenoids and Phenolic Compounds—Evaluation of Varieties With Different Peel Color. Front. Sustain. Food Syst..

[78] Cha K.H., Song D.G., Kim S.M., Pan C.H. (2012). Inhibition of Gastrointestinal Lipolysis by Green Tea, Coffee, and Gomchui (*Ligularia fischeri*) Tea Polyphenols during Simulated Digestion. J. Agric. Food Chem..

[79] Marino M., Del Bo′ C., Tucci M., Venturi S., Mantegazza G., Taverniti V., Møller P., Riso P., Porrini M. (2022). A Mix of Chlorogenic and Caffeic Acid Reduces C/EBPß and PPAR-Γ1 Levels and Counteracts Lipid Accumulation in Macrophages. Eur. J. Nutr..

[80] Vilas-Boas A.A., Oliveira A., Jesus D., Rodrigues C., Figueira C., Gomes A., Pintado M. (2020). Chlorogenic Acids Composition and the Impact of in Vitro Gastrointestinal Digestion on Espresso Coffee from Single-Dose Capsule. Food Res. Int..

[81] Kim I., Moon J.K., Hur S.J., Lee J. (2020). Structural Changes in Mulberry (*Morus microphylla*. Buckl) and Chokeberry (*Aronia melanocarpa*) Anthocyanins during Simulated in Vitro Human Digestion. Food Chem..

[82] Liang L., Wu X., Zhao T., Zhao J., Li F., Zou Y., Mao G., Yang L. (2012). In Vitro Bioaccessibility and Antioxidant Activity of Anthocyanins from Mulberry (*Morus atropurpurea* Roxb.) Following Simulated Gastro-Intestinal Digestion. Food Res. Int..

[83] Khochapong W., Ketnawa S., Ogawa Y., Punbusayakul N. (2021). Effect of in Vitro Digestion on Bioactive Compounds, Antioxidant and Antimicrobial Activities of Coffee (*Coffea arabica* L.) Pulp Aqueous Extract. Food Chem..

[84] Abdelazim Mohdaly A.A., Ramadan M.F. (2022). Characteristics, Composition and Functional Properties of Seeds, Seed Cake and Seed Oil from Different Brassica Carinata Genotypes. Food Biosci..

[85] Alves R.C., Rodrigues F., Nunes M.A.A., Vinha A.F., Oliveira M.B.P.P., Galanakis C. (2017). State of the Art in Coffee Processing By-Products.

[86] Sorolla M.A., Hidalgo I., Sorolla A., Montal R., Pallisé O., Salud A., Parisi E. (2021). Microenvironmental Reactive Oxygen Species in Colorectal Cancer: Involved Processes and Therapeutic Opportunities. Cancers.

[87] Condello M., Meschini S. (2021). Role of Natural Antioxidant Products in Colorectal Cancer Disease: A Focus on a Natural Compound Derived from *Prunus spinosa*, Trigno Ecotype. Cells.

[88] Snezhkina A.V., Kudryavtseva A.V., Kardymon O.L., Savvateeva M.V., Melnikova N.V., Krasnov G.S., Dmitriev A.A. (2020). ROS Generation and Antioxidant Defense Systems in Normal and Malignant Cells. Oxid. Med. Cell Longev..

[89] Rebollo-Hernanz M., Zhang Q., Aguilera Y., Martín-Cabrejas M.A., de Mejia E.G. (2019). Relationship of the Phytochemicals from Coffee and Cocoa By-Products with Their Potential to Modulate Biomarkers of Metabolic Syndrome in Vitro. Antioxidants.

[90] Heeger A., Kosińska-Cagnazzo A., Cantergiani E., Andlauer W. (2017). Bioactives of Coffee Cherry Pulp and Its Utilisation for Production of Cascara Beverage. Food Chem..

[91] Rebollo-Hernanz M., Zhang Q., Aguilera Y., Martín-Cabrejas M.A., Gonzalez de Mejia E. (2019). Phenolic Compounds from Coffee By-Products Modulate Adipogenesis-Related Inflammation, Mitochondrial Dysfunction, and Insulin Resistance in Adipocytes, via Insulin/PI3K/AKT Signaling Pathways. Food Chem. Toxicol..

[92] Kim S.H., Park S.Y., Park Y.L., Myung D.S., Rew J.S., Joo Y.E. (2017). Chlorogenic Acid Suppresses Lipopolysaccharide-Induced Nitric Oxide and Interleukin-1β Expression by Inhibiting JAK2/STAT3 Activation in RAW264.7 Cells. Mol. Med. Rep..

[93] Mehaya F.M., Mohammad A.A. (2020). Thermostability of Bioactive Compounds during Roasting Process of Coffee Beans. Heliyon.

[94] Budryn G., Nebesny E., Oracz J. (2015). Correlation between the Stability of Chlorogenic Acids, Antioxidant Activity and Acrylamide Content in Coffee Beans Roasted in Different Conditions. Int. J. Food Prop..

[95] Gallego-Barceló P., Bagues A., Benítez-Álvarez D., López-Tofiño Y., Gálvez-Robleño C., López-Gómez L., del Castillo M.D., Abalo R. (2023). Evaluation of the Effects of Instant Cascara Beverage on the Brain-Gut Axis of Healthy Male and Female Rats. Nutrients.

[96] Moreno O.A.V., Swarr T.E., Asselin A.C., Milà i Canals L., Colley T., Valdivia S. (2015). Implementation of Life Cycle Management Practices in a Cluster of Companies in Bogota, Colombia. Int. J. Life Cycle Assess..

[97] De Marco I., Riemma S., Iannone R. (2018). Life Cycle Assessment of Supercritical CO_2_ Extraction of Caffeine from Coffee Beans. J. Supercrit. Fluids.

[98] Giraldi-Díaz M.R., De Medina-Salas L., Castillo-González E., León-Lira R. (2018). Environmental Impact Associated with the Supply Chain and Production of Grounding and Roasting Coffee through Life Cycle Analysis. Sustainability.

[99] Brommer E., Stratmann B., Quack D. (2011). Environmental Impacts of Different Methods of Coffee Preparation. Int. J. Consum. Stud..

[100] Rajesh Banu J., Kannah R.Y., Kumar M.D., Preethi, Kavitha S., Gunasekaran M., Zhen G., Awasthi M.K., Kumar G. (2021). Spent Coffee Grounds Based Circular Bioeconomy: Technoeconomic and Commercialization Aspects. Renew. Sustain. Energy Rev..

[101] Overturf E., Pezzutto S., Boschiero M., Ravasio N., Monegato A. (2021). The Circo (Circular Coffee) Project: A Case Study on Valorization of Coffee Silverskin in the Context of Circular Economy in Italy. Sustainability.

[102] Ministerio de Industria y Turismo C. y T. Niveles de Madurez de la Tecnología Technology Readiness Levels. TRLs. Una introducción. https://www.mincotur.gob.es/publicaciones/publicacionesperiodicas/economiaindustrial/revistaeconomiaindustrial/393/notas.pdf.

[103] Bozzola M., Charles S., Ferretti T., Gerakari E., Manson H., Rosser N., von der Goltz P. (2021). The Coffee Guide.

[104] Iriondo-DeHond A., Iriondo-DeHond M., Del Castillo M.D. (2020). Applications of compounds from coffee processing by-products. Biomolecules.

